# Long‐Term Drop‐Seizure Response Patterns to Cannabidiol According to Genetic Status in Lennox–Gastaut Syndrome

**DOI:** 10.1002/cns.71221

**Published:** 2026-11-01

**Authors:** Ji‐Hoon Na, Hee yeon Cho, Hayoon Jeon, Hyunjoo Lee, Young‐Mock Lee

**Affiliations:** ^1^ Department of Pediatrics, Division of Pediatric Neurology, Gangnam Severance Hospital Yonsei University College of Medicine Seoul Republic of Korea

**Keywords:** cannabidiol, drop seizures, Lennox–Gastaut syndrome, long‐term follow‐up, treatment response, whole‐exome sequencing

## Abstract

**Background:**

Lennox–Gastaut syndrome (LGS) is a genetically heterogeneous developmental and epileptic encephalopathy in which variability in long‐term cannabidiol (CBD) response remains incompletely understood. We evaluated whether long‐term drop‐seizure response trajectories to CBD differ according to genetic status.

**Methods:**

This single‐center retrospective longitudinal study analyzed systematically collected clinical follow‐up data from 61 patients with LGS treated with pharmaceutical‐grade CBD; 46 patients with ≥ 24 months of follow‐up were included. Patients were classified as pathogenic/likely pathogenic variant‐positive (PV+) or variant‐negative (PV–). Drop‐seizure reduction trajectories were assessed at 3‐month intervals over 24 months.

**Results:**

Drop‐seizure reduction improved rapidly and was sustained over 24 months in both PV+ and PV– groups, with no significant differences between groups at the prespecified comparison time points. Longitudinal response trajectories showed substantial overlap, and sustained response was observed in 76.1% of patients.

**Conclusion:**

Long‐term drop‐seizure response to CBD in LGS was sustained but not stratified by genetic status, supporting a syndrome‐level treatment approach irrespective of genetic findings.

## Graphical abstract

Cannabidiol was associated with rapid and sustained long‐term reduction of drop seizures in patients with Lennox–Gastaut syndrome over 24 months of follow‐up. Durable treatment responses were observed across genetically heterogeneous patients, with substantial overlap in longitudinal response trajectories regardless of genetic status. These findings support cannabidiol as a broadly applicable long‐term treatment option for drop seizures in Lennox–Gastaut syndrome.

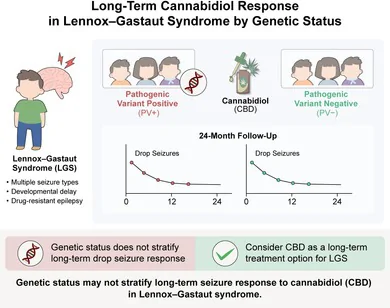

## 1 Introduction

Lennox–Gastaut syndrome LGS is a severe form of developmental and epileptic encephalopathy characterized by multiple drug‐resistant seizure types, including the hallmark tonic type, along with distinctive electroencephalographic abnormalities and persistent cognitive and behavioral impairment. Onset typically occurs in early childhood, with lifelong clinical consequences [1]. LGS imposes a substantial burden on patients, families, and society, as frequent and injurious seizures—particularly drop seizures—contribute significantly to trauma, hospitalizations, and reduced quality of life for patients and caregivers [1, 2]. Despite the availability of multiple antiseizure medications, long‐term seizure freedom remains uncommon, underscoring the need for effective and durable therapeutic strategies [1, 3]. Highly purified cannabidiol (CBD) has emerged as an important adjunctive treatment; randomized controlled trials (RCTs) demonstrate significant reductions in drop‐seizure frequency in patients with LGS, leading to regulatory approval [1, 4]. However, real‐world studies and management‐focused reviews have highlighted considerable interindividual variability in treatment response and retention, particularly over longer follow‐up periods, indicating that factors associated with sustained seizure control in LGS remain incompletely understood [5, 6, 7, 8]. Long‐term observational data suggest that while some patients maintain sustained benefit, others experience fluctuating or attenuated seizure control despite continued therapy, highlighting clinically meaningful heterogeneity beyond initial response [7, 8]. Consensus recommendations emphasize that CBD response is individualized; they recommend judging long‐term effectiveness based on maximum tolerated dose and ongoing clinical course rather than early seizure reduction alone [9].

LGS is increasingly recognized as a genetically heterogeneous developmental and epileptic encephalopathy, reflecting the broader complexity of epilepsy genetics rather than a single etiologic entity [10]. Advances in genetic sequencing have expanded the spectrum of pathogenic variants associated with LGS, encompassing genes involved in ion‐channel function, synaptic signaling, and neurodevelopmental regulation, with considerable interindividual and population‐level variability [10, 11]. Cohort‐based analyses have confirmed that identifiable pathogenic variants are present in a substantial subset of patients with LGS, although no uniform genotype–phenotype correlation has been established [11]. In parallel, experimental and translational studies of cannabinoids in genetic epilepsy models have suggested that seizure‐modifying effects may vary according to underlying genetic mechanisms, yet these observations have rarely been translated into genetically stratified clinical analyses [12, 13]. Consequently, despite growing insights into both epilepsy genetics and cannabinoid‐based therapies, the relationship between genetic status and long‐term CBD response—particularly the durability and trajectory of drop‐seizure control in LGS—remains poorly defined, representing a critical gap in the literature [10, 11, 12, 13].

In light of this gap between genetic characterization and therapeutic outcomes in LGS, longitudinal clinical data provide an opportunity to examine whether treatment response evolves differently across genetically heterogeneous patient groups. Accordingly, we conducted a single‐center retrospective study using systematically collected longitudinal clinical data to describe long‐term drop‐seizure response patterns to CBD over a 24‐month follow‐up period in patients with LGS; additionally, we investigated whether these patterns differed according to genetic status. Rather than focusing on short‐term efficacy or aggregated outcomes, we examined individual‐level trajectories of drop‐seizure response over time. Given the increasing emphasis on stratified and precision‐oriented approaches in LGS management, recent reviews have underscored the need for clinically grounded studies that explore treatment‐response variability in relation to genetic background [14, 15]. This study addresses a clinically relevant gap between genetic insights and real‐world treatment outcomes in LGS.

## 2 Methods

### 2.1 Study Design and Participants

This study was a single‐center retrospective analysis of systematically collected clinical follow‐up data from patients diagnosed with LGS who were treated with CBD at Gangnam Severance Hospital, Yonsei University College of Medicine between 2019 and 2026. The overall study design and predefined longitudinal assessment schedule are summarized in Figure 1. Patients were diagnosed with LGS according to the latest diagnostic criteria, including multiple seizure types with mandatory tonic seizures, characteristic electroencephalographic findings such as slow spike‐and‐wave complexes and generalized paroxysmal fast activity, and early onset epilepsy associated with developmental impairment. Clinical, electroencephalographic, and neurodevelopmental data were reviewed to confirm the diagnosis in all cases [1, 4, 7, 16].

**FIGURE 1:**
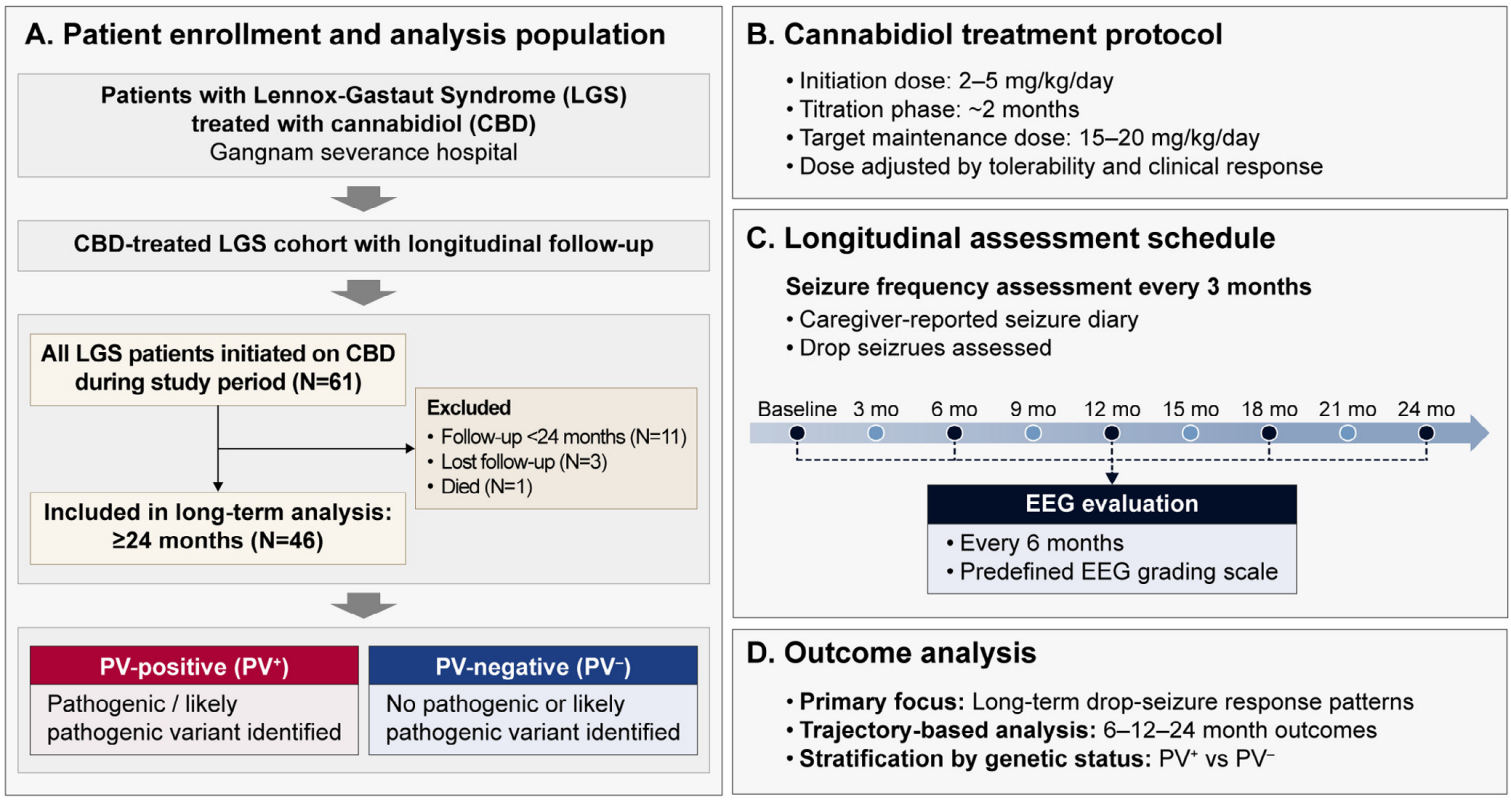
Study design and longitudinal assessment timeline of cannabidiol treatment in Lennox–Gastaut syndrome. This study represents a retrospective analysis of prospectively collected clinical data from patients with Lennox–Gastaut syndrome treated with pharmaceutical‐grade cannabidiol. Patients who completed at least 24 months of follow‐up with available study data were included in the long‐term analysis population. Cannabidiol was initiated and titrated according to a standardized institutional protocol, followed by maintenance therapy. Seizure frequency, including drop seizures, was assessed every 3 months using caregiver‐reported seizure diaries. Electroencephalographic evaluations were performed at baseline and at 6‐month intervals (6, 12, 18, and 24 months) using a predefined EEG grading scale. Long‐term drop‐seizure response trajectories were analyzed over the 24‐month follow‐up period and stratified according to genetic status.

Patients with LGS who initiated CBD treatment during the study period were consecutively enrolled. The long‐term analysis population comprised patients who completed ≥ 24 months of follow‐up after CBD initiation. Patients were excluded if follow‐up duration was < 24 months, CBD treatment was discontinued before the 24‐month evaluation for any reason, or longitudinal seizure data were insufficient for analysis [17]. This cut‐off enabled assessment of long‐term seizure response patterns, but risks retention bias; therefore, long‐term effectiveness estimates should be interpreted accordingly.

All included patients underwent comprehensive genetic evaluation using whole‐exome sequencing. Genetic variants were classified according to established criteria, and patients were categorized based on the presence or absence of pathogenic or likely pathogenic variants for subsequent analyses [10, 11]. Genetic testing was completed prior to or during CBD treatment in all patients. PV+ status was defined as the presence of a pathogenic or likely pathogenic variant identified on genetic testing. PV– status was defined as the absence of identified pathogenic or likely pathogenic variants.

CBD was administered uniformly as a pharmaceutical‐grade oral solution (Epidiolex, 100 mg/mL). Treatment was initiated at a starting dose of 2–5 mg/kg/day and gradually titrated over approximately 2 months according to individual tolerability, with a target maintenance dose of 15–20 mg/kg/day [18, 19]. Dose adjustments were guided by clinical response and adverse events at the discretion of the treating physician, following a standardized institutional protocol. Patients were evaluated at regular 3‐month intervals after CBD initiation [17]. At each visit, seizure frequency, including drop seizures, and treatment tolerability were assessed using caregiver‐reported seizure diaries in conjunction with clinical chart review. Concomitant antiseizure medications were maintained according to routine clinical practice, and changes in background therapy were recorded throughout the follow‐up period. Clobazam and valproate dosing were individualized according to clinical response, tolerability, and routine laboratory monitoring rather than a predefined study protocol. Baseline clobazam dose, baseline valproate dose, and baseline drop‐seizure frequency category were additionally recorded to address potential differences in baseline seizure burden and concomitant medication exposure between genetic subgroups.

### 2.2 Outcome Measures

#### 2.2.1 Seizure Outcomes

Seizure outcomes focused on drop seizures, given their distinct clinical relevance in LGS. For this study, drop seizures were operationally defined as seizure events associated with a sudden loss of postural tone or axial tonic contraction leading to a fall or near‐fall requiring external support, as documented in caregiver seizure diaries and clinical records. Tonic seizures were classified as drop seizures only when accompanied by an objective fall or near‐fall event [4]. Seizure frequency was assessed at 3‐month intervals after initiation of CBD, based on caregiver‐reported seizure diaries reviewed and confirmed during routine outpatient visits.

Because baseline drop‐seizure burden varied substantially across patients, baseline drop‐seizure frequency was categorized as 1–10/day, 11–20/day, or > 20/day. Longitudinal drop‐seizure outcomes were evaluated primarily as relative changes from baseline. Seizure frequency at the time of CBD initiation was used as the reference point, and subsequent outcomes were categorized according to conventional seizure‐reduction ranges: no effect, < 25% reduction, 25%–49% reduction, 50%–74% reduction, 75%–99% reduction, and drop‐seizure freedom. Drop‐seizure freedom was defined as the complete absence of drop seizures during the assessment interval [3, 4].

#### 2.2.2 Electroencephalographic Outcomes

EEG outcomes were assessed at 6‐month intervals as an exploratory secondary electroclinical measure. EEG findings were not considered surrogate markers of cannabidiol efficacy but were evaluated to determine whether longitudinal improvement in drop seizures was accompanied by parallel changes in electroencephalographic findings during routine clinical follow‐up. EEG findings were graded longitudinally using a predefined ordinal scale:

1. normalization.

2. slow and disorganized background rhythm without focal or unilateral sharp‐wave discharges.

3. slow and disorganized background rhythm with focal or unilateral sharp‐wave discharges.

4. slow and disorganized background rhythm with multifocal sharp‐wave discharges.

5. slow and disorganized background rhythm with multifocal sharp‐wave discharges and generalized slow spike‐and‐wave discharges, with or without generalized paroxysmal fast activities.

EEG grades were assigned based on clinical EEG interpretations documented in the medical records. Because EEG acquisition was not standardized across all visits and included both routine EEG studies and prolonged EEG recordings according to clinical indications, EEG findings were analyzed descriptively and interpreted as exploratory observations [3].

#### 2.2.3 Safety and Tolerability Outcomes

Safety and tolerability were evaluated throughout the follow‐up period. Adverse events were recorded based on patient‐ or caregiver‐reported symptoms and clinician‐documented assessments at each visit. Treatment tolerability was further assessed by documenting CBD dose adjustments and treatment discontinuation attributable to adverse events or intolerance, as recorded in the clinical charts.

### 2.3 Analysis Populations

To ensure clarity in outcome reporting, three distinct analysis populations were defined. The safety population included all patients who received at least one dose of CBD. The efficacy population comprised patients with available baseline seizure assessment and at least one post‐baseline follow‐up evaluation. The long‐term analysis population consisted of patients who completed a minimum of 24 months of follow‐up after CBD initiation, allowing assessment of long‐term drop‐seizure response trajectories. Analyses according to genetic status were performed within this long‐term analysis population.

### 2.4 Statistical Analysis

Descriptive statistics were used to summarize demographic, clinical, electroencephalographic, and seizure‐related variables. Continuous variables are presented as median with interquartile range (IQR), and categorical variables are presented as number and percentage. Normality of data distribution was assessed using the Shapiro–Wilk test. As most continuous variables did not follow a normal distribution, non‐parametric methods (Mann–Whitney *U* test) were applied. Biological sex, as recorded in the clinical data, was additionally considered as an exploratory variable. Comparisons of baseline characteristics and longitudinal drop‐seizure outcomes according to sex were performed descriptively using Fisher's exact test for categorical variables and the Mann–Whitney *U* test for continuous variables. Because of the modest sample size and sex imbalance, particularly within subgroups, no formal multifactorial model including sex, genetic status, and their interaction was fitted. Comparisons between PV+ and PV– groups were performed using the Mann–Whitney *U* test for continuous variables and Fisher's exact test for categorical variables, given the sample size and distribution characteristics. Longitudinal drop‐seizure outcomes were analyzed at predefined 3‐month intervals up to 24 months after CBD initiation. Between‐group comparisons of drop‐seizure reduction at specific time points (6, 12, 18, and 24 months) were conducted using the Mann–Whitney *U* test and are presented descriptively. Given the exploratory nature and small sample size, we did not fit a formal repeated‐measures model. No adjustment was made for multiple comparisons, as the analyses were exploratory and hypothesis‐generating. For long‐term response pattern analysis, patients were classified into mutually exclusive response categories based on their longitudinal drop‐seizure reduction trajectories over 24 months. Differences in the distribution of response patterns between PV+ and PV– groups were evaluated using Fisher's exact test, with *p* values reported descriptively.

All statistical analyses were performed using Python (version 3.12; Python Software Foundation) with standard scientific libraries. A two‐sided *p* value of < 0.05 was considered statistically significant, unless otherwise specified.

## 3 Results

### 3.1 Patient Characteristics at Baseline

Sixty‐one patients with LGS underwent CBD treatment at our center during the study period. The following were excluded based on selection criteria: 11 patients who had not yet completed 24 months of follow‐up, 3 patients lost to follow‐up, and 1 patient who died because of an intercurrent medical condition unrelated to cannabidiol treatment. The final cohort comprised 46 patients, including 20 in the PV+ group and 26 in the PV– group. Baseline demographic and clinical characteristics of the long‐term cohort (≥ 24 months of follow‐up) are summarized in Table 1.

**TABLE 1:** Demographic and baseline clinical characteristics of patients with LGS (≥ 24 months of follow‐up).

Characteristic	All patients	PV+	PV–	*p*
Number of patients, *n*	46	20	26	—
Sex, *n* (%)				
Male	33 (71.7%)	15 (75.0%)	18 (69.2%)	0.740
Female	13 (28.3%)	5 (25.0%)	8 (30.8%)	
Age at first seizure, months	10.2 (2.6–22.7)	7.0 (2.4–13.2)	10.3 (3.9–25.3)	0.338
Age at diagnosis of LGS, years	3.8 (2.2–8.4)	3.3 (2.2–8.8)	4.0 (2.5–7.9)	0.830
Age at CBD initiation, years	9.6 (5.4–16.6)	9.6 (5.6–16.2)	9.6 (5.4–15.4)	0.940
Time from LGS diagnosis to CBD initiation, years	1.5 (0.1–8.4)	1.0 (0.1–6.9)	1.8 (0.3–8.8)	0.591
Final CBD dose, mg/kg/day	15.0 (15.0–15.0)	15.0 (15.0–15.0)	15.0 (15.0–15.0)	0.142
ASMs tried prior to CBD, *n*	8.0 (7.0–10.0)	8.5 (7.0–10.3)	7.0 (6.3–9.0)	0.302
Concomitant clobazam use, *n* (%)	46 (100.0%)	20 (100.0%)	26 (100.0%)	1.000
Baseline clobazam dose, mg/day	10.0 (5.0–15.0)	10.0 (5.0–15.0)	10.0 (5.0–20.0)	0.884
Concomitant valproate use, *n* (%)	45 (97.8%)	19 (95.0%)	26 (100.0%)	1.000
Baseline valproate dose, mg/kg/day	20.0 (15.0–25.0)	17.0 (12.7–22.5)	20.1 (18.0–25.0)	0.089
Seizure type at CBD initiation				
Generalized tonic, *n* (%)	44 (95.7%)	19 (95.0%)	25 (96.2%)	1.000
Atonic, *n* (%)	14 (30.4%)	3 (15.0%)	11 (42.3%)	0.105
Generalized tonic–clonic, *n* (%)	25 (54.3%)	15 (75.0%)	10 (38.5%)	0.017
Myoclonic, *n* (%)	26 (56.5%)	11 (55.0%)	15 (57.7%)	1.000
Absence, *n* (%)	7 (15.2%)	2 (10.0%)	5 (19.2%)	0.682
Focal, *n* (%)	22 (47.8%)	11 (55.0%)	11 (42.3%)	0.386
Baseline drop‐seizure frequency				
1–10/day	22 (47.8%)	9 (45.0%)	13 (50.0%)	0.898
11–20/day	21 (45.7%)	10 (50.0%)	11 (42.3%)	
> 20/day	3 (6.5%)	1 (5.0%)	2 (7.7%)	
History of status epilepticus, *n* (%)	4 (8.7%)	4 (20.0%)	0 (0.0%)	0.027

*Note:* Values are presented as median (interquartile range) or number (percentage), as appropriate. Seizure types are not mutually exclusive. Drop seizures were operationally defined as fall/near‐fall‐associated events (tonic or atonic semiology) as described in the Methods.

Abbreviations: ASMs, antiseizure medications; CBD, cannabidiol; IQR, interquartile range; LGS, Lennox–Gastaut syndrome; PV, pathogenic variant; PV–, pathogenic variant‐negative; PV+, pathogenic variant‐positive.

Overall, 33 patients (71.7%) were male and 13 (28.3%) were female. The distribution of sex was comparable between the PV+ and PV– groups (75.0% vs. 69.2% male, *p* = 0.74). The median age at first seizure was 10.2 months (IQR 2.6–22.7), and the median age at diagnosis of LGS was 3.8 years (IQR 2.2–8.4). Patients initiated CBD at a median age of 9.6 years (IQR 5.4–16.6), with a median interval of 1.5 years (IQR 0.1–8.4) from LGS diagnosis to CBD initiation. Prior to CBD treatment, patients had received multiple antiseizure medications (median 8.0, IQR 7.0–10.0). Concomitant clobazam use at CBD initiation was observed in all patients (46/46, 100.0%), whereas concomitant valproate use was observed in 45 patients (97.8%). The median baseline clobazam dose was 10.0 mg/day (IQR 5.0–15.0), with no significant difference between the PV+ and PV– groups (10.0 [5.0–15.0] vs. 10.0 [5.0–20.0] mg/day, *p* = 0.884). The median baseline valproate dose was 20.0 mg/kg/day (IQR 15.0–25.0) and was likewise comparable between the PV+ and PV– groups (17.0 [12.7–22.5] vs. 20.1 [18.0–25.0] mg/kg/day, *p* = 0.089). Baseline drop‐seizure burden was also similar between groups, with most patients experiencing daily drop seizures prior to CBD initiation (Table 1).

At CBD initiation, generalized tonic seizures were present in 95.7% of patients, with similar frequencies in the PV+ and PV– groups; however, only tonic seizures associated with a fall or near‐fall event were classified as drop seizures, as defined earlier. Atonic seizures were observed in 30.4% of the cohort and were more frequent in the PV– group than in the PV+ group (42.3% vs. 15.0%, respectively), although this difference did not reach statistical significance (*p* = 0.105). Generalized tonic–clonic seizures were more prevalent in the PV+ group than in the PV– group (75.0% vs. 38.5%, respectively; *p* = 0.017). Baseline drop‐seizure burden was high in most patients. Drop seizures occurred 1–10 times/day in 22 patients (47.8%), 11–20 times/day in 21 patients (45.7%), and > 20 times/day in 3 patients (6.5%). The distribution of baseline drop‐seizure frequency categories was comparable between the PV+ and PV– groups (*p* = 0.898), with 1–10/day in 9 PV+ patients (45.0%) and 13 PV– patients (50.0%), 11–20/day in 10 PV+ patients (50.0%) and 11 PV– patients (42.3%), and > 20/day in 1 PV+ patient (5.0%) and 2 PV– patients (7.7%). A history of status epilepticus was observed exclusively in the PV+ group (20.0% vs. 0.0%, respectively; *p* = 0.027).

No patient had undergone prior epilepsy surgery, corpus callosotomy, or vagus nerve stimulation implantation, and no such procedures were performed during the 24‐month cannabidiol treatment period.

### 3.2 Genetic Etiologies and Longitudinal Drop‐Seizure Outcomes in PV‐Positive Patients

Table 2 summarizes genetic etiologies and longitudinal drop‐seizure outcomes among PV+ patients with ≥ 24 months follow‐up (*n* = 20). Most genetic etiologies were represented by a single patient, with only a few genes observed in two or more patients, reflecting the genetic heterogeneity of LGS. Drop‐seizure reduction was assessed at 6, 12, 18, and 24 months after CBD initiation. Across genetic categories, drop‐seizure reduction generally increased over time, with substantial reductions observed by 12 months in many patients and sustained responses through 24 months.

**TABLE 2:** Genetic etiologies and longitudinal drop‐seizure outcomes in PV‐positive patients (*n* = 20).

Gene (no. of patients)	Age at diagnosis of LGS, years	Drop‐seizure reduction 6 months, %	Drop‐seizure reduction 12 months, %	Drop‐seizure reduction 18 months, %	Drop‐seizure reduction 24 months, %	EEG outcome (baseline→12 months), *n*		EEG outcome (baseline→24 months), *n*	
						Improved	Unchanged	Improved	Unchanged
***CLCN4* (3)** ^a^	5.3	100.0	100.0	100.0	100.0	1	2	2	1
***SCN2A* (2)** ^a^	5.4	37.5	75.0	75.0	75.0	1	1	1	1
***SHANK3* (2)** ^a^	4.4	50.0	62.5	55.0	55.0	0	2	1	1
*ASNS* (1)	2.0	75.0	100.0	100.0	100.0	1	0	1	0
*CDG* (1)	2.7	100.0	100.0	100.0	100.0	1	0	1	0
*CDKL5* (1)	2.7	50.0	100.0	100.0	100.0	1	0	1	0
*PAFAH1B1* (1)	3.5	10.0	10.0	50.0	25.0	0	1	0	1
*SCN1A* (1)	2.1	75.0	100.0	100.0	100.0	1	0	1	0
*SCN2B* (1)	2.2	25.0	100.0	100.0	100.0	1	0	1	0
*SLC19A3* (1)	9.6	100.0	100.0	100.0	100.0	1	0	1	0
*TSC2* (1)^a^	9.5	100.0	100.0	100.0	100.0	0	1	0	1
*WDR62* (1)	29.3	100.0	100.0	100.0	100.0	1	0	1	0
**1q43q44 deletion (1)**	4.7	75.0	100.0	100.0	100.0	1	0	1	0
**m.15728 C>T (1)** ^a^	13.1	50.0	75.0	100.0	100.0	1	0	0	1
**m.8993T>G (1)**	2.2	75.0	90.0	100.0	100.0	0	1	1	0
**Xp22.13 deletion (1)**	2.2	75.0	100.0	100.0	100.0	0	1	1	0

*Note:* Drop‐seizure reduction values are reported at 6, 12, 18, and 24 months after cannabidiol initiation. EEG outcomes are reported as change from baseline to 12 months and 24 months using the predefined EEG grading scale. Because baseline EEG grade was 5 in all patients, “Unchanged” indicates no change in EEG grade, whereas “Improved” indicates a decrease in EEG grade to ≤ 4 at follow‐up. The a denotes patients in whom EEG grade remained unchanged despite clinically meaningful improvement in drop seizures (≥ 50% reduction), reflecting potential dissociation between electroencephalographic grading and clinical seizure outcomes. Because EEG acquisition was not standardized across all visits and included both routine EEG studies and prolonged EEG recordings according to clinical indications, EEG outcomes are presented descriptively and should be interpreted as exploratory electroclinical observations.

Abbreviations: CBD, cannabidiol; EEG, electroencephalography; LGS, Lennox–Gastaut syndrome; mo, months; pts., patients; PV, pathogenic variant.

^a^ EEG grade remained unchanged at follow‐up despite ≥ 50% reduction in drop seizures at 24 months.

At 6 months, drop‐seizure freedom (100% reduction) was observed in 8 of 20 patients (40.0%). At 12 months, 14 of 20 patients (70.0%) achieved drop‐seizure freedom. At 24 months, 15 of 20 patients (75.0%) achieved drop‐seizure freedom. Among genes represented by more than one patient, all three patients with *CLCN4* pathogenic variants achieved drop‐seizure freedom by 24 months. Five patients did not achieve drop‐seizure freedom at 24 months, including patients with *SCN2A* (*n* = 2), *SHANK3* (*n* = 2), and *PAFAH1B1* (*n* = 1) pathogenic variants.

EEG outcomes were summarized as changes from baseline to 12 and 24 months. At baseline, all patients demonstrated generalized slow spike‐and‐wave activity and generalized paroxysmal fast activity, corresponding to EEG Grade 5. EEG outcomes were categorized as improved or unchanged based on the predefined EEG grading scale. Among patients with available EEG data, improvement or persistence of EEG abnormalities without grade change was commonly observed at both 12 and 24 months. Notably, in several patients, EEG grade remained unchanged at follow‐up despite clinically meaningful improvement in drop seizures (≥ 50% reduction at 24 months; Table 2).

### 3.3 Longitudinal Drop‐Seizure Response Trajectories According to Genetic Status

Figure 2 illustrates longitudinal drop‐seizure response trajectories to cannabidiol over a 24‐month follow‐up period according to genetic status. Median drop‐seizure reduction increased rapidly in both PV+ and PV– groups within the first 6 months after CBD initiation and was generally sustained thereafter (Figure 2A). Early response trajectories during the initial 6‐month period were comparable between the two groups. At 6, 12, 18, and 24 months, no statistically significant differences in median drop‐seizure reduction were observed between PV+ and PV– groups (all *p* > 0.05).

**FIGURE 2:**
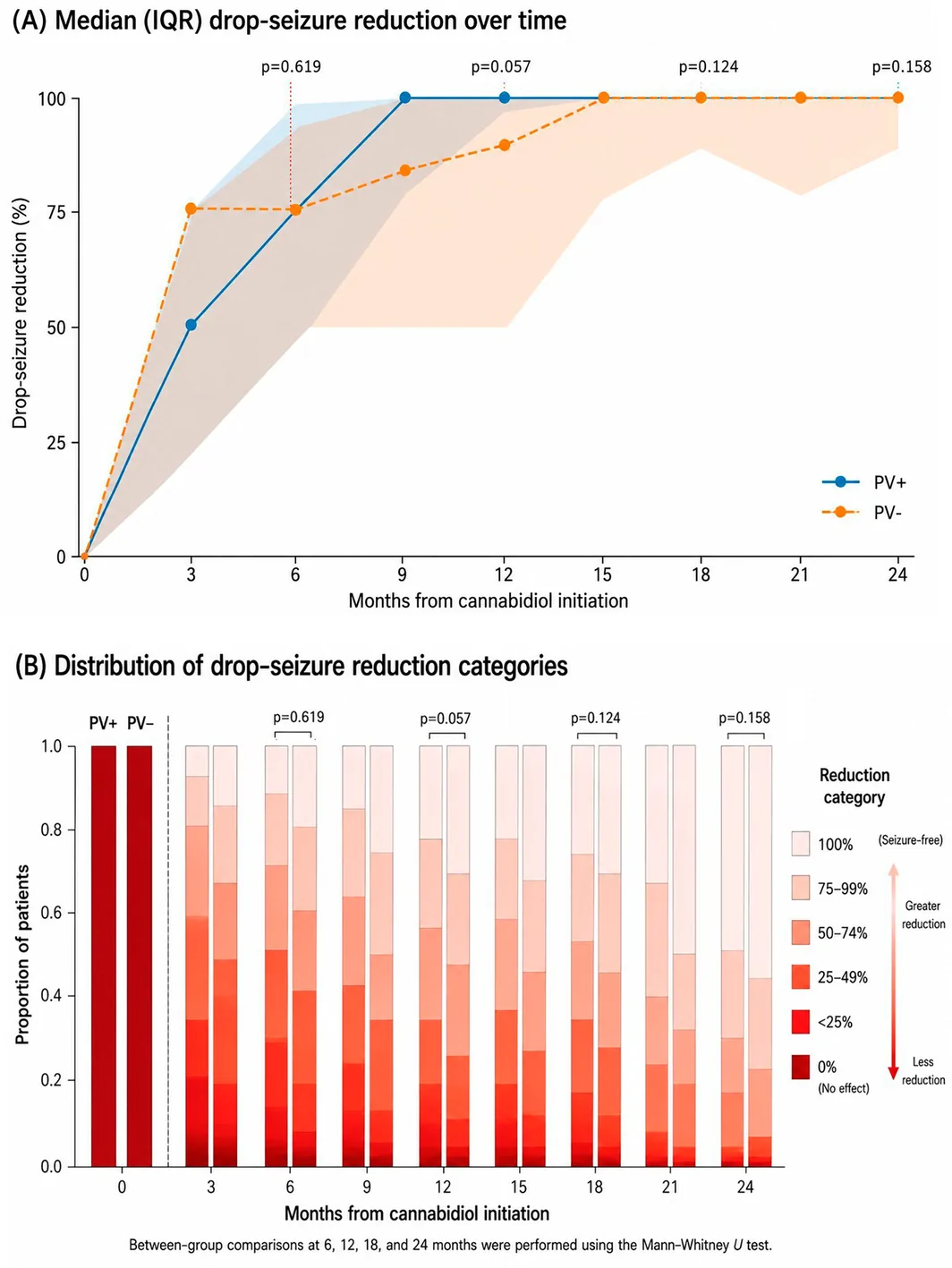
Longitudinal drop‐seizure response trajectories to cannabidiol according to genetic status in LGS. (A) Median (interquartile range) drop‐seizure reduction over time in patients with pathogenic variants (PV+) and those without pathogenic variants (PV–). Drop‐seizure reduction was assessed at 3‐month intervals up to 24 months after cannabidiol initiation. Between‐group comparisons at 6, 12, 18, and 24 months were performed using the Mann–Whitney *U* test. *p* values are presented for descriptive comparisons at prespecified time points and were not adjusted for multiple comparisons. (B) Distribution of drop‐seizure reduction categories at each time point in PV+ and PV− groups. Reduction categories were defined as no effect, < 25% reduction, 25%–49% reduction, 50%–74% reduction, 75%–99% reduction, and drop‐seizure freedom. Categories are represented using a single‐hue color gradient, with darker shades indicating lower reduction and lighter shades indicating greater reduction. Between‐group comparisons at 6, 12, 18, and 24 months were performed using the Mann–Whitney *U* test. CBD, cannabidiol; IQR, interquartile range; LGS, Lennox–Gastaut syndrome; PV, pathogenic variant; PV+, pathogenic variant‐positive; PV–, pathogenic variant‐negative.

Analysis of the distribution of drop‐seizure reduction categories further demonstrated substantial overlap between PV+ and PV– groups across all assessed time points (Figure 2B). At each follow‐up visit, both groups showed a progressive shift toward higher reduction categories, with an increasing proportion of patients achieving near‐complete or complete drop‐seizure control over time. Overall distribution patterns were comparable between PV+ and PV– groups at 6, 12, 18, and 24 months, without evidence of distinct separation according to genetic status. In exploratory analyses, no significant differences in drop‐seizure reduction were observed between male and female patients at any time point.

### 3.4 Long‐Term Drop‐Seizure Response Pattern Distribution According to Genetic Status

Long‐term drop‐seizure response patterns over the 24‐month follow‐up period are summarized in Table 3. Sustained responders constituted the majority of the cohort (35 of 46 patients, 76.1%), including 17 of 20 PV+ patients (85.0%) and 18 of 26 PV– patients (69.2%). Furthermore, 4 patients (8.7%) demonstrated a delayed response, all of whom were in the PV– group (15.4% of PV– patients), whereas no delayed responses occurred in the PV+ group. Partial response was seen in 6 patients (13.0%), with similar proportions in the PV+ and PV– groups (10.0% vs. 15.4%, respectively). Only one patient (2.2%) met criteria for non‐response at 24 months, and this patient belonged to the PV+ group.

**TABLE 3:** Long‐term drop‐seizure response pattern distribution according to genetic status.

Response pattern	All patients (*n* = 46)	PV+ (*n* = 20)	PV– (*n* = 26)	*p*
Sustained responders	35 (76.1%)	17 (85.0%)	18 (69.2%)	0.302
Delayed responders	4 (8.7%)	0 (0.0%)	4 (15.4%)	0.121
Partial responders	6 (13.0%)	2 (10.0%)	4 (15.4%)	0.684
Non‐responders	1 (2.2%)	1 (5.0%)	0 (0.0%)	0.435

*Note:* Response patterns were defined based on longitudinal drop‐seizure reduction trajectories over the 24‐month follow‐up period. Sustained responders were defined as patients with ≥ 75% drop‐seizure reduction maintained at both 12 and 24 months. Delayed responders were defined as patients with < 75% reduction at 6 months who subsequently achieved ≥ 75% reduction at 12 or 24 months. Partial responders were defined as patients with 25%–74% reduction at 24 months. Non‐responders were defined as patients with < 25% reduction at 24 months. *p* values were calculated using Fisher's exact test and are provided for descriptive purposes only.

Abbreviations: PV, pathogenic variant; PV–, pathogenic variant–negative; PV+, pathogenic variant–positive.

Across all response categories, no significant differences in distribution were observed between PV+ and PV– groups (all *p* > 0.05). Overall, the distribution of long‐term drop‐seizure response patterns demonstrated substantial overlap between the two groups, without evidence of distinct separation according to genetic status.

### 3.5 Safety and Tolerability

Safety and tolerability outcomes were assessed throughout the follow‐up period. No unexpected safety signals emerged during long‐term CBD treatment. Mild adverse events were observed in some patients: mild diarrhea (*n* = 3), mild elevation of liver enzymes (*n* = 2), and mild somnolence (*n* = 5). These adverse events were managed with gradual dose titration and symptomatic adjustment and improved over time. Importantly, none led to permanent treatment discontinuation or affected the final maintenance dose of CBD. Given the focus of this analysis on long‐term drop‐seizure response patterns, safety outcomes were not further quantified. Liver enzyme abnormalities were mild and clinically manageable, and somnolence generally improved with observation and individualized medication adjustment.

## 4 Discussion

In this 24‐month longitudinal analysis of patients with LGS treated with CBD, long‐term drop‐seizure response trajectories did not differ significantly according to genetic status. As shown in Figure 2, both PV+ and PV– groups exhibited rapid early improvement followed by sustained seizure reduction, with substantial overlap in median trajectories and response distributions. Consistent with these longitudinal distributions, trajectory‐based classification of long‐term drop‐seizure response (Table 3) also demonstrated comparable patterns between PV+ and PV– patients, without clear separation according to genetic status. The absence of apparent genetic stratification in long‐term outcomes should be interpreted cautiously, given the modest sample size and etiologic heterogeneity of the cohort; nevertheless, the observed overlap in longitudinal response trajectories suggests that PV status alone did not clearly separate long‐term CBD‐associated drop‐seizure response in this cohort.

From a clinical perspective, the overlapping long‐term drop‐seizure trajectories in PV+ and PV– groups indicate that CBD can be considered at the syndrome level in LGS, rather than restricting its use based on genetic status alone [18, 20]. This finding aligns with long‐term real‐world and extension studies showing sustained antiseizure effects of CBD across heterogeneous epilepsy populations, including those with diverse genetic etiologies [17, 18]. Importantly, the absence of clear genetic stratification in long‐term outcomes should not be interpreted as a lack of therapeutic specificity, but rather as a reflection of the distributed and network‐based nature of seizure generation in LGS, which has been emphasized in contemporary treatment algorithms [20]. Experimental and translational data further support this framework, demonstrating that CBD exerts pleiotropic effects on neuronal and glial signaling, neuroinflammation, and synaptic transmission, thereby modulating shared epileptic networks rather than single‐gene mechanisms [21]. Clinical observations in monogenic epilepsies similarly suggest that responsiveness to CBD is not confined to specific genetic subtypes, reinforcing its role as a broadly applicable adjunctive therapy [17]. Together, these considerations provide reassurance to clinicians that CBD may be used for long‐term management of drop seizures in LGS irrespective of PV status, while underscoring the importance of longitudinal follow‐up beyond early response when counseling patients and caregivers [18, 20]. Although total seizure outcomes were also recorded, the present study intentionally focused on drop seizures because they represent a core and clinically consequential seizure type in LGS and can be quantified more reliably than heterogeneous seizure‐type combinations.

In this cohort, CBD produced large and consistent long‐term effects on drop seizures, with most patients achieving substantial and sustained reduction over 24 months, a finding that aligns with the recognized clinical burden of drop seizures and their contribution to morbidity in LGS [22, 23]. Notably, despite a higher baseline severity signal in the PV+ group—reflected by a significantly greater history of status epilepticus prior to CBD initiation—long‐term drop‐seizure trajectories remained comparable between PV+ and PV– patients, suggesting that higher pre‐treatment severity did not preclude robust clinical benefit from CBD [23, 24]. Clinically, we did not observe recurrent status epilepticus during follow‐up among PV+ patients; however, given the observational design and limited sample size, this finding should be interpreted cautiously and cannot be directly attributed to CBD treatment [22, 23]. At the same time, overlapping response patterns between PV+ and PV– groups do not negate the potential relevance of precision‐oriented hypotheses within selected genetic subgroups [25]. In this context, the uniformly favorable response in patients with *CLCN4* pathogenic variants (all three achieving drop‐seizure freedom by 24 months) warrants further investigation in larger cohorts [25]. Although the small number of patients precludes definitive conclusions, this observation may warrant further investigation into potential gene‐specific influences on CBD response in larger cohorts [22, 25].

Following the aforementioned clinical patterns, our findings also highlight a dissociation between EEG changes and clinical response in LGS, in which improvement in drop seizures with CBD was not always accompanied by an immediate change in EEG grade [3, 16]. This observation is consistent with the historical and electroclinical characterization of LGS, where EEG abnormalities reflect widespread and persistent network dysfunction rather than a direct, short‐term correlate of seizure frequency [16]. In particular, generalized paroxysmal fast activity (GPFA)—closely associated with tonic and drop seizures—may decrease or disappear with effective treatment, whereas diffuse background slowing and generalized slow spike‐and‐wave activity often persist, thereby anchoring patients within the same ordinal EEG grade despite meaningful clinical improvement [3, 26]. This phenomenon is reflected in Table 2, which captures patients who achieved substantial drop‐seizure reduction at 24 months despite unchanged EEG grade, emphasizing that EEG grading may lag behind clinical stabilization [3, 26]. Emerging evidence from chronic thalamocortical recordings further supports this interpretation, demonstrating that electrophysiologic markers in LGS represent dynamic network states that may evolve more slowly than observable seizure outcomes [27]. Taken together, these data underscore the importance of interpreting EEG findings longitudinally and in conjunction with clinical trajectories, rather than as surrogate markers of cannabidiol treatment response. Furthermore, because EEG acquisition was not standardized with respect to recording duration and included both routine and prolonged EEG studies, these findings should be regarded as exploratory observations rather than definitive measures of treatment efficacy [16, 27].

In extending the electroclinical dissociation described above, the present findings align with prior RCTs and subsequent real‐world studies evaluating CBD in LGS. Pivotal RCTs established the short‐term efficacy of CBD in reducing drop seizures, and long‐term open‐label extension studies further demonstrated that these benefits can be sustained, supporting its role as a long‐term adjunctive therapy [28, 29]. However, these studies were primarily designed to assess overall responder rates at predefined time points and did not specifically address individual‐level longitudinal trajectories or potential differences according to genetic status [28, 29]. Evidence from broader real‐world cohorts similarly suggests sustained effectiveness of CBD across diverse epilepsy populations and clinical settings, although such studies often included heterogeneous diagnoses and variable follow‐up intervals, limiting their ability to characterize syndrome‐specific long‐term trajectories in LGS [7, 17]. Although the genetic heterogeneity of LGS has been increasingly characterized at the population level, prior genotype‐focused investigations have mainly described variant distributions rather than treatment‐related trajectories over time [11]. Experimental and translational studies in genetic epilepsy models further highlight mechanistic diversity but do not directly inform durable clinical response patterns in LGS [12]. Within this existing literature, the present study adds a complementary perspective by integrating uniform 24‐month follow‐up with genetic stratification to examine long‐term drop‐seizure trajectories in PV+ and PV– patients, thereby helping to bridge short‐term efficacy data with real‐world considerations relevant to sustained CBD treatment in LGS.

Taken together with the longitudinal patterns observed in this cohort, the present findings suggest that genetic status alone did not appear to meaningfully differentiate durable drop‐seizure outcomes with CBD in LGS [30]. From a clinical perspective, these data support a syndrome‐level approach in which genotype informs etiologic understanding and prognosis, but does not necessarily serve as a sole determinant for CBD use when sustained control of drop seizures is the therapeutic goal [30]. We additionally explored sex as a variable and did not observe a clear sex‐associated difference in long‐term drop‐seizure response; however, the relatively small number of female patients limits definitive conclusions.

Several limitations should be considered when interpreting these results. First, this was a single‐center study with a modest sample size, which limits the generalizability of the findings and indicates that all analyses were exploratory in nature. The sample size was determined by available patients meeting the prespecified 24‐month follow‐up criterion; therefore, the study was not formally powered to detect modest differences between genetic subgroups. Because of this, repeated time‐point comparisons were not adjusted for multiple testing, as prespecified in the Methods, and *p* values should be interpreted descriptively rather than as confirmatory evidence [30]. Second, although CBD dosing was guided by widely used weight‐based titration protocols in routine practice, actual exposure at a given mg/kg dose can vary substantially both between and within individuals [31]. This variability introduces uncertainty into the relationship between prescribed dose and systemic drug levels; however, such variability is biologically plausible given known differences in CBD metabolism and metabolite profiles, as well as the influence of food intake on absorption, and consequently identical doses may yield different systemic concentrations across patients [31, 32]. In addition, EEG findings were evaluated using a simplified grading system derived from routine clinical practice rather than a validated quantitative EEG biomarker. Therefore, EEG changes should be interpreted as exploratory electroclinical observations rather than direct measures of cannabidiol treatment response. Future studies incorporating therapeutic drug monitoring and more standardized administration conditions may therefore help refine dosing strategies and clarify how pharmacokinetic variability relates to long‐term clinical trajectories in LGS [31, 33].

## 5 Conclusions

In this 24‐month longitudinal study of patients with LGS treated with CBD, long‐term drop‐seizure trajectories and response patterns showed substantial overlap between PV+ and PV– groups, indicating that genetic status alone did not meaningfully stratify durable clinical outcomes. These data suggest that a syndrome‐level treatment perspective may be appropriate, in which CBD can be considered for long‐term management of drop seizures irrespective of PV status. Future studies with larger, multicenter cohorts and integrated biomarker approaches, including pharmacokinetic and electrophysiologic measures, will be essential to refine predictors of sustained response and to advance precision‐oriented care in LGS [34].

## Author Contributions

Ji‐Hoon Na: conceptualization; investigation; methodology; formal analysis; visualization; project administration; resources; writing – original draft preparation; writing – review and editing. Hee yeon Cho: data curation. Hayoon Jeon: data curation. Hyunjoo Lee: resources. Young‐Mock Lee: conceptualization; writing – review and editing. All authors provided approval of the final version for publication and agree to be accountable for all aspects of the work.

## Funding

The authors have nothing to report.

## Ethics Statement

The study protocol was reviewed and approved by the Institutional Review Board of Gangnam Severance Hospital, Yonsei University College of Medicine (approval number: 3‐2022‐0135). We confirm that we have read the Journal's position on issues involved in ethical publication and affirm that this report is consistent with those guidelines.

## Consent

Written informed consent was obtained from patients or their legal guardians, as appropriate, in accordance with institutional requirements. All data were anonymized prior to analysis to ensure patient confidentiality.

## Conflicts of Interest

The authors declare no conflicts of interest.

## Data Availability

The data that support the findings of this study are available on request from the corresponding author. The data are not publicly available due to privacy or ethical restrictions.
